# Congenital transmission of *Trypanosoma cruzi* in Argentina, Honduras, and Mexico: study protocol

**DOI:** 10.1186/1742-4755-10-55

**Published:** 2013-10-11

**Authors:** Pierre Buekens, Maria-Luisa Cafferata, Jackeline Alger, Fernando Althabe, José M Belizán, Yves Carlier, Alvaro Ciganda, Eric Dumonteil, Rubi Gamboa-Leon, Elizabeth Howard, Maria Luisa Matute, Sergio Sosa-Estani, Carine Truyens, Dawn Wesson, Concepcion Zuniga

**Affiliations:** 1 School of Public Health and Tropical Medicine, Tulane University, 1440 Canal Street, Ste. 2430, New Orleans, Louisiana LA 70112, USA; 2 Unidad de Investigación Clínica y Epidemiológica Montevideo (UNICEM), Montevideo, Uruguay; 3 Instituto de Enfermedades Infecciosas y Parasitología Antonio Vidal, Tegucigalpa, Honduras; 4 Hospital Escuela, Facultad de Ciencias Medicas, UNAH, Tegucigalpa, Honduras; 5 Instituto de Efectividad Clínica y Sanitaria (IECS), Buenos Aires, Argentina; 6 Université Libre de Bruxelles (ULB), Brussels, Belgium; 7 Universidad Autónoma de Yucatán, Centro de Investigaciones Regionales “Dr. Hideyo Noguchi”, Mérida, Mexico; 8 National Laboratory, Ministry of Health, Tegucigalpa, Honduras; 9 Instituto Nacional de Parasitología “Dr. Mario Fatala Chaben”-ANLIS, Buenos Aires, Argentina; 10 National Chagas Program, Ministry of Health, Tegucigalpa, Honduras

**Keywords:** *Trypanosoma cruzi*, Chagas disease, Congenital transmission, Latin America

## Abstract

**Background:**

*Trypanosoma cruzi* has been divided into Discrete Typing Units I and non-I (II-VI). *T. cruzi* I is predominant in Mexico and Central America, while non-I is predominant in most of South America, including Argentina. Little is known about congenital transmission of *T. cruzi* I. The specific aim of this study is to determine the rate of congenital transmission of *T. cruzi* I compared to non-I.

**Methods/design:**

We are conducting a prospective study to enroll at delivery, 10,000 women in Argentina, 7,500 women in Honduras, and 13,000 women in Mexico. We are measuring transmitted maternal *T. cruzi* antibodies by performing two rapid tests in cord blood (Stat-Pak, Chembio, Medford, New York, and Trypanosoma Detect, InBios, Seattle, Washington). If at least one of the results is positive, we are identifying infants who are congenitally infected by performing parasitological examinations on cord blood and at 4–8 weeks, and serological follow-up at 10 months. Serological confirmation by ELISA (Wiener, Rosario, Argentina) is performed in cord and maternal blood, and at 10 months. We also are performing *T. cruzi* standard PCR, real-time quantitative PCR and genotyping on maternal venous blood and on cord blood, and serological examinations on siblings. Data are managed by a Data Center in Montevideo, Uruguay. Data are entered online at the sites in an OpenClinica data management system, and digital pictures of data forms are sent to the Data Center for quality control. Weekly reports allow for rapid feedback to the sites.

**Trial registration:**

Observational study with ClinicalTrials.gov Identifier NCT01787968

## Background

Chagas’ disease, or American trypanosomiasis, is caused by the protozoan parasite *Trypanosoma cruzi*. It is a major cause of morbidity and mortality in the Americas, and an estimated 9 million persons are currently infected [1]. Of particular concern is the fact that mothers with Chagas’ disease can transmit *T. cruzi* to their fetuses [2].

Mother-to-child transmission of *T. cruzi* has all the characteristics required to be a public health priority, as it is relatively frequent, severe, identifiable, and treatable [3]. In reality, it is a neglected disease and a missed opportunity. Therefore, it is urgent to better understand the epidemiology of mother-to-child transmission and to develop effective prevention programs.

*T. cruzi* has been divided into Discrete Typing Units (DTUs): *T. cruzi* I (TcI) and *T. cruzi* Non-TcI (II-VI). In humans, TcI is predominant in Mexico and Central America, while Non-TcI is predominant in most of South America, including Argentina [4]. In recent studies from Argentina, the risk of congenital transmission has been estimated to vary between 2.6% and 7.9% [5-8]. By contrast, we know very little about the congenital transmission of TcI. It has been suggested that congenital transmission of *T. cruzi* is strain related [2,9], and there is an urgent need to know if TcI transmits differently than Non-TcI. Our primary hypothesis is that congenital transmission rates are different for TcI versus Non-TcI.

*T. cruzi* infected women living in regions where TcI is predominant could differ in terms of risk factors for transmitting *T. cruzi* to their offspring. Low maternal age, low parity, HIV/AIDS, exposure to vectors and high parasitic load have been reported as risk factors for congenital *T. cruzi* infection [9-14]. Family clustering has been observed in Argentina [15], suggesting that some mothers might be predisposed to transmit repeatedly.

There is limited information about the perinatal outcomes of infected infants in low endemicity areas. Congenital *T. cruzi* infection has been associated with premature rupture of the membranes and preterm births, in addition to neonatal complications [14]. Further studies are needed to document the impact of *T. cruzi* infection on perinatal outcomes.

The specific aims of this study are:

– 1. To determine the rate of congenital transmission of TcI compared to Non-TcI;

– 2. To compare the *T. cruzi* infected mothers’ characteristics and exposure to vectors in regions where TcI is predominant and regions where Non-TcI is predominant; and

– 3. To describe the birth outcomes of infected and uninfected infants born to TcI and Non-TcI seropositive women.

## Methods/design

### Overview

**Figure 1:**
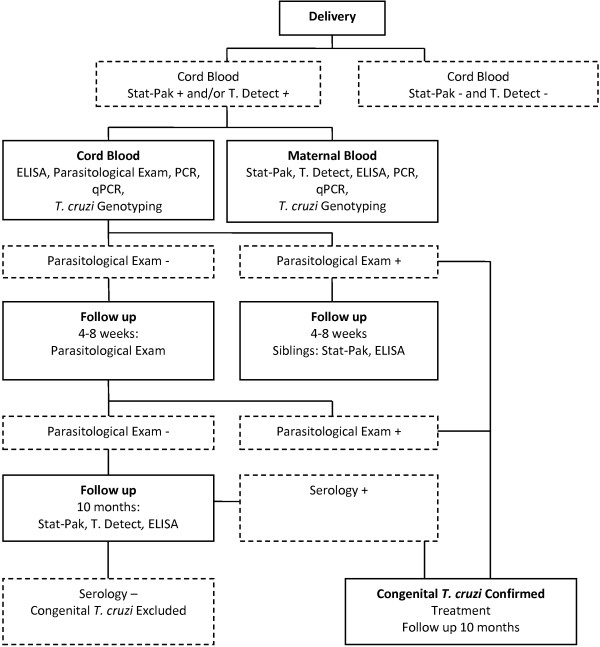
Study design.

We are conducting a prospective study in Mexico, Honduras, and Argentina. We are enrolling women at delivery, collecting umbilical cord blood to measure antibodies of maternal origin, and examining and following up the infants of seropositive mothers and siblings (Figure 1).

At the time of delivery and as soon as possible thereafter, umbilical cord venous blood is collected for all deliveries during the time periods selected for the study. Written informed consent is obtained immediately after delivery (informed consent during labor and delivery is not ethically acceptable). If the woman declines to participate, the cord blood sample is discarded, and no data are recorded. If the woman accepts to participate, we are measuring transmitted maternal *T. cruzi* antibodies by performing two rapid tests in cord blood (Stat-Pak, Chembio, Medford, New York, and Trypanosoma Detect, InBios, Seattle, Washington). If at least one of the results is positive, we are identifying infants who are congenitally infected by performing parasitological examinations on cord blood and at 4–8 weeks, and serological follow-up at 10 months. Serological confirmation by recombinant ELISA (Wiener, Rosario, Argentina) is performed in cord and maternal blood, and at 10 months. We will perform PCR followed by a qPCR and genotyping on the blood of infected mothers and on the umbilical cord blood.

### Variables

The exposure to TcI will be defined by *T. cruzi* genotyping showing TcI on maternal blood and/or cord blood. In the absence of successful *T. cruzi* genotyping on maternal or cord blood, births occurring in Mexico or Honduras will be considered exposed to TcI.

The exposure to Non-TcI will be defined by *T. cruzi* genotyping showing Non-TcI on maternal blood and/or cord blood. In the absence of successful *T. cruzi* genotyping on maternal or cord blood, births occurring in Argentina will be considered exposed to Non-TcI.

The exposure to both TcI and Non-TcI (co-infection) will be defined by *T. cruzi* genotyping of both TcI and Non-TcI on maternal blood and/or on cord blood, or of TcI on maternal blood and Non-TcI on cord blood, or of Non-TcI on maternal blood and TcI on cord blood.

The primary outcome (Specific Aim 1) will be the congenital *T. cruzi* infection, which will be defined as the presence of one or more of the following criteria:

– Presence of parasites in cord blood (direct parasitological microscopic examination); and/or

– Presence of parasites in infant’s blood at 4–8 weeks (direct parasitological microscopic examination); and/or

– Persistence of *T. cruzi*-specific antibodies at 10 months, as measured by two tests.

A seropositive mother will have at least one positive rapid test on cord blood and a positive ELISA on maternal blood.

The mothers’ characteristics and exposure to vectors variables (Specific Aim 2) that will be collected include:

– Maternal age;

– Parity (including current birth);

– Infected sibling (yes/no) (siblings previously treated for *T. cruzi* infection will be presumed as having been infected);

– Maternal parasitic DNA amount;

– HIV serology (according to tests performed routinely in each country);

– Presence of vectors in the household (Infestation) (yes/no).

– Location in an area with risk of vectorial transmission (yes/no)

The birth outcomes (Specific Aim 3) to be measured are the following:

– Premature rupture of the membranes (PROM), defined as rupture of membranes before admission or before the onset of contractions, whatever the timing of the rupture; [14]

– Gestational age (completed weeks), based on the Last Menstrual Period (LMP) or a clinical estimate (including ultrasound, if available) [16]. In the absence of an ultrasound, the clinical estimate will be based on the Capurro Score [17]. This score is the standard method to evaluate gestational age by physical examination of an infant at birth in Argentina and is widely used in Latin America. The clinical estimate will be used if the discrepancy between the LMP-based estimate and the clinical estimate is greater than 10 days; a preterm birth is <37 weeks;

– Birthweight (grams); a low birthweight baby weighs <2,500 g;

– Head circumference (cm);

– Length (cm);

– Neonatal complications: We will use the definitions of Torrico et al. [14] for the following variables, to be registered at a physical examination of the infant at not later than 24 hours of life:

– Hepatomegaly (when ≥ 2 cm below the right costal margin);

– Splenomegaly (whatever the spleen size under the left costal margin);

– Anasarca (palpebral, genital, or leg edema alone will not be considered);

– Petechiae (whatever their localization);

– Respiratory Distress Syndrome (RDS) will be considered when at least one of the following signs is present: tachypnea, throbbing of the ala nasi, expiratory grunting, intercostal retraction, facial or systemic cyanosis (peripheral cyanosis will not be considered);

– Neurological signs will be classified as present or absent based on tone, level of alertness, Moro and other primary neonatal reflexes, deep tendon reflexes, spontaneous motor activity, pupil diameter (in search of mydriasis or miosis), bulging of fontanelles, and convulsions.

Data about birthweight of siblings and breastfeeding are collected by interviewing the mother at home using the Honduras Demographic and Health Survey (DHS) questions [18]. The DHS is a standardized survey administered around the world, and it includes validated questions and allows for international comparisons [19]. For each newborn and his/her siblings, we will also collect data about blood transfusions, organ transplants, and previous screening and treatment for *T. cruzi*.

Participating hospitals will be collecting data for all women who consent to participate, using the format of the Perinatal Information System of PAHO/WHO already in use in Argentina, Honduras, and Mexico [20]. The variables collected include maternal age, reproductive history, HIV status, LMP, ultrasound examination, PROM, mode of delivery, multiple births, Apgar score at one and five minutes, birthweight, head circumference, and length.

A live birth is a baby born with any sign of life, irrespective of gestational age [16]. A neonatal death is defined as death before 28 complete days of life [16]. An infant death will be defined as death before one year of life [16].

### Study population

Participating sites include: Hospital Materno Infantil and Hospital General Valladolid, both in Yucatán, México; Hospital Enrique Aguilar Cerrato, La Esperanza in Intibucá, Honduras; Hospital Santa Bárbara in Santa Bárbara, Honduras; and Instituto Maternidad Provincial Nuestra Señora de las Mercedes in Tucumán, Argentina.

All consecutive live births occurring during the defined study period will be included, irrespective of the gestational age or the route of delivery (vaginal or cesarean).

All subjects must meet all of the inclusion criteria to participate in this study.

• *Inclusion criteria***:** Women 18 years old or more, informed consent, live birth.

All subjects meeting any of the exclusion criteria at baseline will be excluded from study participation.

• *Exclusion criteria***:** Women residing outside of the follow-up area.

### Follow-up

Newborns will be followed up at 4–8 weeks and 10 months. In each country, a dedicated team will perform home visits. The follow-up will be done within the following geographical limits: in Mexico, the Yucatan peninsula (i.e., the states of Yucatan, Campeche, and Quintana Roo); in Honduras, the departments of Intibucá and Santa Barbara; and in Argentina, the province of Tucuman. A first home visit will take place at 4–8 weeks postpartum, when parasitological tests will be performed again if the first parasitological examination on cord blood was negative. Those infants with positive tests will be considered infected and referred for treatment. If the newborn has not been discharged from the hospital at four weeks postpartum, the first follow-up visit will be done in the hospital, and the first home visit will be done within four weeks after discharge to collect data on siblings. Those siblings who are not tested during the first home visit will receive a second visit at 10 months postpartum.

The mothers of confirmed infected children and/or mothers who are themselves confirmed as seropositive will be informed and advised to contact their local health provider. Repeated household visits will be performed until follow-up at the health provider is confirmed, and until the treatment of the infant is completed. Every effort will be made to ensure that infants with congenital *T. cruzi* infection identified at birth or at the 4-to-8-week visit are treated by the health providers before the 10-month follow-up visit and that infants diagnosed at the 10-month follow-up visit are also treated without delay. Newborns and infected siblings will be treated according to local standards, which include a course of 30 to 60 days of benznidazole or nifurtimox [2]. In Honduras and Mexico, and in public hospitals in Argentina, the treatment can only be obtained from the Ministry of Health. We will provide each infected subject with a follow-up card, which will be stamped and signed by the health provider at each follow-up visit and when treatment is given. The mother will also be asked to complete the card on a daily basis to indicate when treatment has been given. We will perform household visits every two weeks to confirm treatment. During household visits, we will collect information from the subjects’ cards and take digital pictures (with date and time) of the cards. We will also register the number of doses of benznidazole or nifurtimox made available to the subject and take digital pictures (with date and time) of the drug supply available in the household.

Geographic data will be collected for vector risk analysis. Street addresses are often unreliable in the areas where participating women live; thus, detailed information will be collected at enrollment. During the first home visit, considerable effort will be made to locate the household, involving key informants if needed. Once located, we will use handheld computers with GPS to map the location of the household. We will use established procedures and software developed by the Centers for Disease Control and Prevention (CDC) [21]. The latitude and longitude of the main entrance are determined after obtaining the appropriate GPS signal, and additional characteristics of the household are noted. The sampling procedures included in the CDC software are not needed here, but the system allows us to navigate back to the household for return visits*.* In addition, these GPS coordinates will be entered into a GIS database (ArcView Geographic Information Systems [ArcGIS] software 9.3, Environmental Systems Research Institute, Redlands, CA) to produce maps of the geographic distribution of seropositive cases. These maps will be used to estimate vectorial exposure in the households from the seropositive cases.

### Entomological data

Entomological data will be obtained from two different sources to estimate vectorial exposure in the households from seropositive mothers. First, we will use vector distribution and house infestation data from the respective vector surveillance and control programs in Argentina and Honduras, which provide geocoded information and maps on the presence of vectors of different species at the village/locality level. For each household of a seropositive mother, we will thus determine if it is located in a village with or without known house infestation by triatomines using cumulated vector data from the past five years. For the Yucatan Peninsula, Mexico, in the absence of vector control programs, we will use published entomologic risk maps [22]. Based on these maps, households located in villages with low or absent risk will be classified as without house infestation, and households from villages with medium or high risk will be classified as with house infestation.

Second, we will use the community participation method, which was successfully piloted in and previously used in Argentina as well [23]. All of the households from seropositive mothers will be included, corresponding to an estimated 100 households in Mexico, 300 in Honduras, and 200 in Argentina. During the first household visit, information on Chagas’ disease and triatomines will be provided, including photographs and the display of dried specimens of the vector, to ensure that study participants will recognize the vector. Participants will then be instructed to collect any triatomines observed inside (domestic) or outside (peri-domestic) of their homes using a safe procedure and to place them into separate labeled plastic vials or bags. Any insects captured since the time of the initial visit will be gathered during the second visit at 10 months. Triatomine species will be identified by local entomologists from vector control programs in Argentina and Honduras and by entomologist researchers in Mexico, using established entomological keys [24,25].

Because vector control is well underway in Argentina and Honduras, we expect house infestation by triatomines to be limited in these countries, while triatomine collections in Mexico will be much more important. Based on these collections, each household will be classified as infested or non-infested, and infested households will be considered at risk of vectorial transmission, irrespective of the *T. cruzi* infection status of the bugs. *T. cruzi* infection status in triatomines will not be determined at this stage, as it is highly unlikely that a sufficient sample size may be reached to determine significant differences in infection rates and *T. cruzi* transmission risk between households. However, all collected insects will be stored in ethanol for subsequent analysis and PCR diagnostic of infection if needed [26,27].

### Blood samples and laboratory evaluations

Immediately after the clamping and sectioning of the umbilical cord, 20 ml of venous umbilical cord blood will be collected from the placental side of the cord with a needle and syringe and placed in EDTA (15 ml) and heparinized (5 ml) tubes following an established protocol. After obtaining informed consent, rapid serological tests will be performed on cord blood within the next six hours. If both rapid serological tests are negative, the mother will be excluded from the study and we will select a randomized sample of the umbilical cord blood samples to be stored.

If one of the cord blood rapid serological tests is positive, parasitological examinations will be performed immediately, and samples will be stored for further testing (10 ml on EDTA will be stored for molecular testing and 5 ml for further serological testing). 15 ml of venous maternal blood will be collected in EDTA tubes from mothers and also stored (10 ml for molecular testing and 5 ml for further serological testing).

The samples to be stored for further molecular testing will be immediately mixed with the same volume of guanidine-HCl 6M, EDTA 0.1 M (pH 8), and kept at 4°C until processed. This standard blood treatment contributes to the stabilization of the sample’s DNA content and improves its long-term conservation as well as favors the parasitic DNA dispersion into the entire sample. Blood samples for further serological tests will be stored in part on filter paper kept at 4°C until use, and additional plasma samples will be frozen at -20°C until use.

During the 4-to-8-week follow-up household visits, 2 ml of blood will be collected in heparinized tubes from the infant by venipuncture, or 6 heparinized capillary tubes will be collected by heel stick and transported to the laboratory in mobile refrigerators for parasitological examination within a maximum of six hours. In those cases in which it is not possible to collect enough blood to fill 6 capillary tubes, collecting a blood sample large enough to fill one capillary tube will be considered acceptable. Finger pricks will be used for siblings. A half drop of blood (10 μl) will be collected from the finger with a disposable pipette for the rapid serological test. An additional 100 μl (5 drops) of blood will be stored on filter paper for ELISA study.

During the 10-month household visit, 3 ml of blood will be collected in EDTA tubes from the infant by venipuncture and transported to the laboratory in mobile refrigerators. Rapid tests will be performed, and blood will be stored for further testing (filter paper at 4°C and plasma at -20°C). In those cases in which venipuncture cannot be performed, we will collect blood by heel prick or digital puncture in an Eppendorf (or equivalent) EDTA tube to perform rapid tests and further testing, and one filter paper for ELISA testing will be considered an acceptable minimum.

The member of the follow up team who will perform the venipunctures will be a certified phlebotomist. We estimate that less than 1% of the samples will be unusable. Table 1 summarizes the blood samples to be collected and analyzed.

**Table 1:** Procedures with blood samples

**Time of extraction**	**Source**	**Blood sample**	**Tests to perform**	**Storage requirements**
Birth	Umbilical cord	15 ml EDTA	Stat-Pak.	-
			T. Detect,	
			ELISA*,	Filter paper 4°C, Plasma -20°C*
			PCR*	10 ml Guanidine 4°C*
	Umbilical cord	5 ml heparinized	Parasitological examination*	Max 6 hours at 4°C
Before hospital discharge*	Maternal, venous in all (+) subjects	15 ml EDTA	Stat-Pak,	-
			T. Detect,	
			ELISA,	Filter paper 4°C, Plasma -20°C,
			PCR	10 ml Guanidine 4°C
Follow-up visit at 4–8 weeks after birth*	Infant, venous, or heel stick	2 ml heparinized, 6 capillary tubes (ideally), or at least one capillary tube	Parasitological examination	Max 6 hours at 4°C
	Siblings, finger prick	10 μl	Stat-Pak	-
		100 μl	ELISA	Filter paper 4°C
Follow-up visit at 10 months after birth*	Infant, venous or heel stick or digital puncture	3 ml EDTA, or at least one Eppendorf EDTA tube and one filter paper	Stat-Pak,	-
			T. Detect,	
			ELISA	Filter paper 4°C, Plasma -20°C

*If at least one rapid test on cord blood is positive.

We will use the Chagas Stat-Pak, a rapid immunochromatographic screening test for detection of anti-*T. cruzi* antibodies in whole blood [28,29]. We validated its use on cord blood in the three countries in which we are working [30]. A WHO comparative evaluation also found 94% sensitivity for the Stat-Pak [31]. Other studies have reported similar results in Bolivia [32-34], but a lower sensitivity in Peru [33]. A half drop of blood (10 μl) is collected with a disposable pipette and placed on the rapid test cassette, and buffer is added. The presence of anti-*T. cruzi* antibodies in the sample produces a pink/purple line (positive result) in the reactive area, whereas no line appears in the absence of the antibodies (negative result). A second pink/purple line in the control area confirms that the reaction was completed and that the test is validated. Reading the results in the appropriate area of the device is performed by recording the absence of any line as negative or a strong or weak line as positive. All Stat-Pak results have to be read within 15 minutes. Digital pictures (with date and time) are taken at the 15-minute mark to document the reading.

In addition to the Chagas Stat-Pak, we will use Trypanosoma Detect (T. Detect) rapid immunochromatographic screening tests (InBios, Seattle, Washington), for detection of anti-*T. cruzi* antibodies in whole blood. T. Detect has a 99% sensitivity and specificity compared to consensus panel sera [35]. The test will be performed according to the manufacturer’s instructions. Briefly, 20 μl of whole blood is added to the test strip. Three drops of chase buffer solution are added into a test tube or assay well where the test strip is placed. All T. Detect results have to be read in 10 minutes. The test is positive when both a control line and test line appear. Digital pictures are taken at the 10-minute mark to document the reading.

Blood stored on filter paper and plasma samples will be analyzed using a commercial ELISA prepared with recombinant *T. cruzi* antigens (Chagatest ELISA recombinant, version 3.0, Wiener Laboratories, Rosario, Argentina), according to the manufacturer protocol. Spots of blood will be punched from the filter paper, eluted in the laboratory, and centrifuged and analyzed. The use of filter paper for ELISA has been previously described [36,37].

A confirmatory ELISA will be performed for all 10-month follow-up tests that are positive or in question. The samples will be sent to Tulane University. Serologic analysis will be performed according to the manufacturer protocol using the FDA-cleared Hemagen Chagas Kit (Hemagen Diagnostics, Inc., Columbia, Maryland), a qualitative ELISA designed for the detection of circulating antibodies to *T. cruzi* in serum and plasma.

Congenital infection with *T. cruzi* is sought for by direct microscopic examination of blood buffy coat collected in six centrifuged microhematocrit heparinized tubes as previously described [2,14,38,39]. In those cases in which it is not possible to collect enough blood to fill 6 capillary tubes, collecting blood to fill at least one capillary tube will be considered acceptable. The detection limit of such parasitological detection was estimated to be 40 parasites/ml (p/ml) [38,39], which allowed the detection of more than 90% of the congenital cases in Bolivian endemic areas [40]. The number of positive microhematocrit tubes containing parasites, as well as the number of detected parasites per tube, allowed estimation of parasitemia as either low (≥ 40 p/ml - < 150 p/ml), medium (≥ 150 p/ml - ≤ 400 p/ml), or high (> 400 p/ml). Positive parasitological examinations will be filmed when possible. The films will be downloaded to the Chagas study webpage and shared between the study sites.

We will perform molecular tests to estimate the parasitic DNA amount (PCR followed by a qPCR in positive cases) and to identify the *T. cruzi* genotype (TcI and/or Non-TcI) on the blood of infected mothers. We will also perform PCR followed by genotyping on the umbilical cord blood samples. An international consensus has recognized direct parasitological examinations of cord or neonatal blood collected in capillary tubes, as well as serological investigations of infant blood at 10 months of age, as the best tools for the biological diagnosis of *T. cruzi* congenital infection, whereas PCR must still be standardized and validated for such use [2]. Thus, the research team is using blood parasitological examinations and late serology to identify infected newborns. PCR will not be used for diagnostic purposes, but it will aid in verifying the presence of parasitic DNA in samples before they are quantified via qPCR or before genotyping.

### Data management

Data will be collected on paper case report forms (CRFs) designed specifically for the study. Data will be collected prospectively and kept confidential. The data will be collected in the local language using hard copy forms, with only the individual study identification number on each form. No subject names will appear in the forms.

For each participating woman, a study number (alpha-numeric, with check digit) will be assigned. A set of labels with each number is designed to be pasted on the CRFs of the mother and her children. Bar-coded labels (Brady, Milwaukee, Wisconsin) will be pasted on each of the blood samples. Names and other personal identifiers are recorded in the inclusion form header, which are stripped from the main body containing data. Each part is identified with a study label number. The inclusion form body will be sent to the local data center for data entry. With this system, it is ensured that personal identifiers will not be taken from the hospital and will be kept securely by the hospital coordinator.

Digital pictures (with date and time) of each CRF and the rapid tests used for screening will be taken in each country by the Field Research Coordinator and regularly sent encrypted to the Data Center by uploading the information to a cloud-based system (Dropbox) [41]. This system allows for a digital backup of all CRFs, as well as a parallel second data entry of 10% of the forms at the Data Center to detect systematic errors at data entry.

CRFs will also be entered on a daily basis in a secure web data management system called OpenClinica, which is open source software for clinical research studies using distributed data entry. This software has been adopted by the Department of Reproductive Health and Research of the World Health Organization as their data management systems, among other public research groups. The system was adapted (forms and rules) specifically for this study by the Clinical and Epidemiological Research Montevideo Unit (UNICEM, Montevideo, Uruguay), the study data center. The data forms, checks, and rules were designed by the study researchers in collaboration with the Data Center.

After the CRF has been entered into OpenClinica, it will be placed into a folder labeled with the participant’s unique subject ID. CRFs will be stored at the research unit’s data entry center in locked cabinets. Only the Country Data Manager has access to documents linking patient personal information to study ID code numbers.

The Country Data Manager in each country will send weekly reports to the Data Center regarding the screening, recruitment, consent, laboratory and follow-up processes of the study. The Data Center will prepare weekly reports about each site and send them to the Country Data Manager and to the Principal Investigator of the study.

### Sample size

We are planning to study 600 mothers with *T. cruzi* infection. The sample size will be achieved by recruiting 13,000 women in Mexico (estimated seroprevalence of 1% in Merida and 0.4% in Valladolid), 7,500 in Honduras (estimated seroprevalence of 4%), and 10,000 in Argentina (estimated seroprevalence of 2%). Based on our pilot study, we estimate that 20% of deliveries will be excluded because the age of the mother will be <18 or because consent is not obtained. In addition, we estimate that 5% of the women will be ineligible because they live outside of the follow-up area in Mexico (which includes the entire Yucatan Peninsula) and that 10% will live outside of the follow-up areas in Honduras and Argentina (which include only each hospital’s department or province).

We estimate that we will recruit 300 mothers with TcI infection and 300 mothers with Non-TcI infection. We will have more than 80% power to detect a difference of 1% transmission for TcI versus 5% for Non-TcI. We estimate that the loss to follow-up will be lower than 10%.

### Analysis plan

We will compute the rate of congenital transmission among seropositive mothers according to the exposure to TcI or Non-TcI with the corresponding confidence intervals and compare them using binomial exact tests. A seropositive mother will have at least one positive rapid test on cord blood and a positive ELISA on maternal blood. Cases with TcI/Non-TcI co-infection and with maternal HIV/AIDS will be analyzed separately, and transmission rates will be compared to the rest of the population.

In a second step, we will repeat the analysis after excluding infants with negative parasitological examinations and negative PCR on cord blood, and with a history of transfusion and/or organ transplant, and/or who are living in households exposed to the vector (presence of vectors and/or location in an area at risk of vector transmission). The resulting congenital transmission rate will be a more conservative estimate, which will be less likely to be biased by non-congenital routes of transmission. We will also stratify according to breastfeeding (yes/no), another potential route of neonatal transmission.

We will adjust the comparison of TcI/Non-TcI transmission rates for potential confounders:

– Maternal age at delivery (in five-year categories);

– Parity (primiparae/multiparae);

– Presence of vectors in the household (yes/no);

– Maternal parasitic DNA amount;

– Mode of delivery (C. section vs. vaginal); and

– Infected sibling (yes/no) (siblings previously treated for *T. cruzi* infection will be presumed as having been infected).

Mantel-Haenszel chi-square tests will be used for stratified analyses, which will take one categorical factor into account at a time. A logistic regression will be also performed and will include all potential confounders. Adjusted odds ratios (OR) and confidence intervals will be estimated.

We will describe birth outcomes (PROM, gestational age, birthweight, head circumference, length, and neonatal complications) of infected and uninfected infants by calculating proportions and confidence intervals for categorical variables (including preterm birth and low birthweight) and means and standard deviations for continuous ones. In a next step, we will stratify the analysis by DTU (TcI/Non-TcI). We will compare intrauterine growth restriction (IUGR) rates. A newborn will be classified as IUGR if its birthweight is below the tenth percentile of the sex-specific birthweight for gestational age curves proposed by Williams et al. [42] for single and multiple births. These growth curves are recommended by the WHO as reference patterns for international comparisons [43,44]. We will use the same reference for the three countries to allow for comparison among sites. In addition, we will also compute IUGR rates according to a more recent U.S. reference curve [45] for single live births to facilitate comparisons with the U.S. literature. We will also analyze birthweight data of infected and uninfected siblings by calculating proportions of low birthweight and “very small babies” and confidence intervals, mean birthweights, and standard deviations.

We will use STATA Version 8.0 [46] software for data analysis.

### Ethical aspects

The described protocol with informed consent and assent for children between 7 and 17 years old was approved by the Institutional Review Board of Tulane University and the ethics committees of the respective collaborating institutions in Argentina, Honduras, and Mexico.

## Abbreviations

CDC: Centers for Disease Control and Prevention; CRF: Case Report Form; DHS: Demographic and Health Survey; DTU: Discrete Typing Units; EDTA: Ethylenediaminetetraacetic Acid; ELISA: Enzyme-linked Immunosorbent Assay; GIS: Geographic Information Systems; GPS: Global Positioning System; IUGR: Intrauterine Growth Restriction; NIAID: National Institute of Allergy and Infectious Diseases; NIH: National Institutes of Health; PAHO: Pan American Health Organization; PCR: Polymerase Chain Reaction; PROM: Premature Rupture of Membrane; qPCR: Quantitative real-time Polymerase Chain Reaction; TcI: *Trypanosoma cruzi* DTU I; WHO: World Health Organization.

## Competing interests

The authors declare that they have no competing interests.

## Authors’ contributions

The work presented here was carried out in collaboration between all authors. PB defined the research theme and designed the first protocol. All other authors contributed to the writing of the final version of the protocol. All authors have read and approved the final manuscript.
